# *In situ* identification of toxin-producing *Clostridioides difficile* in stool samples based on single-cell Raman spectroscopy

**DOI:** 10.3389/fcimb.2025.1556536

**Published:** 2025-05-19

**Authors:** Baodian Ling, Fangsheng Wang, Heli Wu, Yushan Huang, Junyun Huang

**Affiliations:** ^1^ Department of Laboratory Medicine, The First Affiliated Hospital of Gannan Medical University, Ganzhou, Jiangxi, China; ^2^ Department of Gastroenterology, The First Affiliated Hospital of Gannan Medical University, Ganzhou, Jiangxi, China; ^3^ Department of Scientific Research, The First Affiliated Hospital of Gannan Medical University, Ganzhou, Jiangxi, China

**Keywords:** *Clostridioides difficile* (CD), *Clostridioides difficile* infection (CDI), single cell Raman spectroscopy (SCRS), *Clostridioides difficile* toxin, stools

## Abstract

*Clostridioides difficile* (CD) has emerged as one of the most prevalent nosocomial infections in hospitals and is the primary causative agent of antibiotic-associated diarrhea and pseudomembranous colitis. In recent years, *C. difficile*-induced infections have resulted in significant morbidity and mortality worldwide, with a particularly rapid increase in incidence observed in China. *C. difficile* strains are categorized into toxin-producing and non-toxin-producing based on their ability to synthesize toxins, with the pathogenicity of *C. difficile* being strictly dependent on the protein toxins produced by the toxin-producing strains. Therefore, early and rapid identification of toxin-producing *C. difficile* is crucial for the diagnosis and prevention of *Clostridioides difficile* infection (CDI). Currently, the detection methods of *C. difficile* infection carried out by clinical laboratories in China mainly include *C. difficile* toxin-producing culture, cell culture toxin assay, toxin assay by immunological methods, glutamate dehydrogenase (GDH) assay and nucleic acid amplification assay.However, current detection methods for CDI in clinical laboratories in China exhibit significant limitations, such as being time-consuming, operationally complex, and lacking in specificity and sensitivity. Raman microspectroscopy has been shown to have the potential for rapid and reliable identification in microbial diagnostics, with the method reducing the time to results to less than 1 hour, including the processing of clinical samples, the measurement of single-cell Raman spectra, and the final diagnosis through the use of training models. In this study, we aimed to predict *in situ* strain identification and virulent strain identification of 24 raw clinical stool samples by constructing a reference single-cell Raman spectroscopy (SCRS) database of common intestinal flora and *C. difficile*, as well as a reference SCRS database of toxin-producing and non-toxin-producing *C. difficile* strains. The results showed that the accuracy of *C. difficile* strain identification in clinical stool samples was 83%, and the accuracy of virulent strain prediction was 80%. These findings suggest that Raman spectroscopy may be a viable method for the rapid *in situ* identification of virulent and non-virulent *C. difficile* strains and holds promise for clinical application in the rapid diagnosis of CDI.

## 1 Introduction

*Clostridioides difficile* (CD) is an anaerobic, Gram-positive clostridial bacillus (Antonelli et al., 2020). It is the primary pathogen responsible for antibiotic-associated diarrhea and pseudomembranous enteritis (Greenhill, 2010; Young and Hanna, 2014). The main clinical manifestation of *Clostridioides difficile* infection (CDI) is diarrhea, which is non-specific and can be self-limiting in mild cases. However, severe cases can present with pseudomembranous enterocolitis, toxic megacolon, intestinal perforation, and septicemia (Andersson and Hughes, 2014; Knight et al., 2015).*C. difficile* strains are classified into toxin-producing and non-toxin-producing categories. The toxin-producing strains harbor the tcdA and/or tcdB genes, which encode enterotoxin A and cytotoxin B (Geric et al., 2004). These toxins induce inflammatory responses and degradation of intestinal epithelial cells, leading to pseudomembrane formation (Carter et al., 2012; Rupnik and Janezic, 2016). As a conditionally pathogenic bacterium, *C. difficile* can cause severe infectious colitis and has high morbidity and mortality rates worldwide (Huang et al., 2009). Over the past decade, the incidence of CDI in China has shown a rapid increase. CDI is strictly dependent on the protein toxins produced by toxin-producing *C. difficile* (Slimings and Riley, 2014). Therefore, the rapid identification of toxin-producing strains and early diagnosis of CDI are of paramount importance for the effective diagnosis, treatment, and prevention of the disease.

The diagnosis of CDI is based on clinical symptoms (diarrhea without other identifiable causes) and the detection of toxin-producing *C. difficile* in stool samples using various microbiological examination methods. Currently, clinical laboratories in China primarily utilize methods such as *C. difficile* toxin-producing culture, cell culture toxin assays, immunological toxin assays, glutamate dehydrogenase (GDH) assays, and nucleic acid amplification tests. However, these methods have obvious limitations (Koya et al., 2019). *Clostridioides difficile* is difficult to culture and has a long period of time with low sensitivity; cytotoxicity assay is complicated, time-consuming and expensive (Planche and Wilcox, 2011); enzyme-linked immunoassay is not sensitive enough (Perelle et al., 1997; Carter et al., 2007) and is easily interfered with by the quality of specimens (Sakamoto et al., 2018); glutamate dehydrogenase (GDH) assay is usually used as a primary screening test for CDI diagnosis in the laboratory (Crobach et al., 2016);PCR method is specific, sensitive and fast, but it is difficult to be widely used under resource-limited conditions because of its cumbersome operation steps, dependence on expensive equipment and the need for specialized personnel to operate it. Additionally, the complex composition of stool samples can easily interfere with test results. Consequently, the development of novel, rapid, simple, accurate, and sensitive methods for detecting *C. difficile* is of paramount importance for the effective diagnosis of CDI.

Raman scattering was discovered by the Indian physicist Chandrasekhara Venkata Raman (C. V. Raman) in 1928 (Raman and Krishnan, 1928). This technique allows for the analysis of molecular structures based on the vibrational and rotational information of scattered spectra (Mosier-Boss, 2017; Tao et al., 2017). In recent years, Raman spectroscopy (RS) has rapidly developed in the field of microbiology as an emerging method for the identification of bacterial infections.RS is a rapid, non-destructive, label-free biochemical phenotyping technique (Huang et al., 2004; Huang et al., 2010; Li et al., 2012). It provides information on the unique molecular fingerprints of bacteria (Kanno et al., 2021), including nucleic acids, proteins, carbohydrates, lipids, and pigments (Flemming and Wingender, 2010), which are used to characterize the genotypes, phenotypes, and physiological states of microorganisms, thus identifying the microbial samples with a high degree of specificity (Kirchhoff et al., 2018; Ho et al., 2019; Xu et al., 2019; Uysal Ciloglu et al., 2020; Wang et al., 2021). Its labeling-free feature reduces sample destruction and complex pre-processing steps, while the detection process is rapid, usually within minutes, making it suitable for rapid screening and real-time monitoring (Xu et al., 2023). In addition, Raman spectroscopy requires less sample volume and only a trace amount of sample to be analyzed, which is particularly suitable for precious or limited samples, and is non-destructive, so that the sample can be retained after detection for subsequent analysis or research. Its wide applicability enables it to be applied to a wide range of sample forms, such as liquid and solid (Kloß et al., 2015; Liu et al., 2023; Xu et al., 2023), showing strong adaptability.

**Figure 1:**
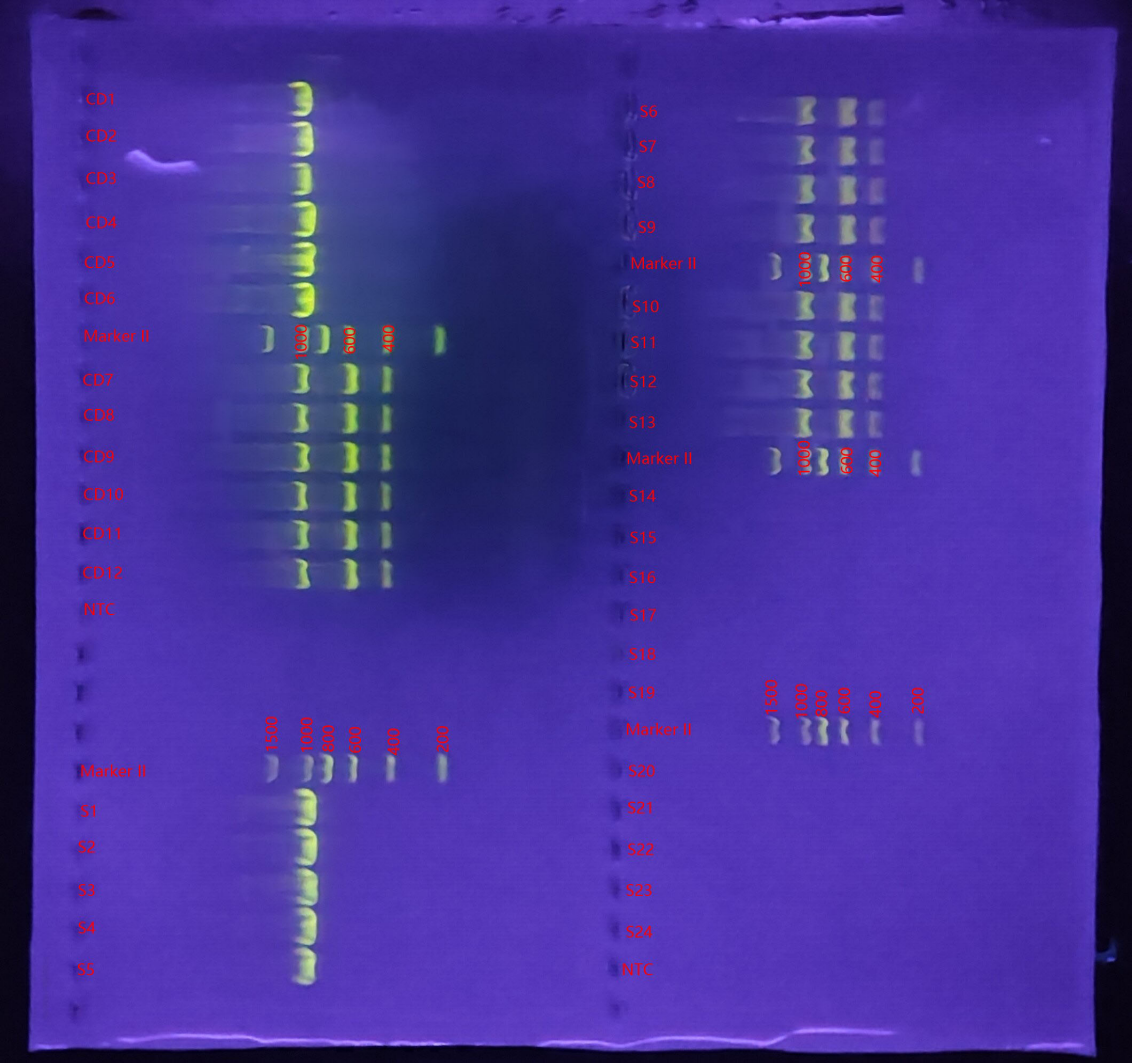
Genetic mapping of *Clostridioides difficile* toxins in selected *C. difficile* strains and fecal specimens. CD, *Clostridioides difficile*; S, stool; NTC, negative control; Marker:6 Each stripe is 200,400,600,800,1000,1500bp;S1-S13:CD+; S14-S24:CD-; S1-S5:tcdA-, tcdB-; S6-S13:tcdA+, tcdB+; CD1-CD6:tcdA-, tcdB-; CD7-CD12:tcdA+, tcdB+.

In this study, we screened 24 raw stool samples from clinical sources, confirmed by multiplex PCR, including 8 samples containing toxin-producing *C. difficile*, 5 containing non-toxin-producing *C. difficile*, and 11 without *C. difficile*. Additionally, 12 C*. difficile* strains were isolated and cultured from these clinical samples, comprising 6 toxigenic and 6 non-toxigenic strains (see **Figure 1**). By establishing a single-cell Raman spectroscopy (SCRS) database for *C. difficile*, we identified *C. difficile in situ* within the stool samples using Raman spectroscopy, distinguishing between toxin-producing and non-toxin-producing strains. The results demonstrated that the bacterial classification accuracy was 100% for isolated samples, 83% for clinical samples, 85% for isolated samples of toxin-producing and non-toxin-producing strains, and 80% for clinical samples of toxin-producing and non-toxin-producing strains. This study indicates that SCRS, with the assistance of deep learning algorithms, can achieve rapid and accurate identification of bacterial strains and effectively differentiate between virulent and non-virulent strains. This capability facilitates the rapid diagnosis, screening, and treatment of CDI, thereby mitigating its spread and enhancing public health.

## 2 Materials and methods

### 2.1 Strains and stool samples

Ten strains of common intestinal bacteria and 12 strains of *C. difficile* were collected from the First Affiliated Hospital of Gannan Medical University. All strains were identified using MALDI-TOF MS (Bruker, Germany), as listed in **Table 1**. The *C. difficile* strains included 6 toxin-producing strains harboring the tcdA and/or tcdB genes, and 6 non-toxin-producing strains. The presence of the toxin A (tcdA) and toxin B (tcdB) genes in *C. difficile* was confirmed using multiplex PCR. Bacterial DNA from the *C. difficile* strains was extracted using the heat-excitation method, followed by amplification of the tcdA and tcdB toxin genes. The PCR cycles were performed under the following conditions: initial denaturation at 94°C for 5 minutes, followed by 32 cycles of denaturation at 94°C for 30 seconds, annealing at 56°C for 30 seconds, extension at 72°C for 1 minute, and a final extension at 72°C for 10 minutes. The PCR products were separated by agarose gel electrophoresis at 2.0% (180V, 80mA, 18 minutes), and the results were subsequently analyzed. The primers were obtained from S. Persson et al (Persson et al., 2008), and those used for the identification of *C. difficile* toxin genes were provided by Shanghai Sangong Bioengineering Co.

**Table 1:** Strain list.

Serial number	Bacterial strain	Serial number	Bacterial strain
1	*Escherichia coli*	7	*Lactobacillus casei*
2	*Enterococcus faecalis*	8	*Bacteroides faecis*
3	*Klebsiella pneumoniae*	9	*Clostridium* sp*orogenes*
4	*Enterobacter cloacae*	10	*Clostridium perfringens*
5	*Staphylococcus epidermidis*	11-22	*Clostridioides difficile*
6	*Enterococcus faecium*		

1-10: Ten most common gut colonizing bacterial strains; 11-22: Twelve *Clostridioides difficile* strains (including 6 toxigenic and 6 non-toxigenic strains).

Stool samples with suspected *C. difficile* infection were collected and cultured on *C. difficile* selective medium CCFA. Yellow colonies with rough surfaces and uneven edges were selected for strain identification using MALDI-TOF MS. Simultaneously, the toxin A (tcdA) and toxin B (tcdB) genes of *C. difficile* were determined by multiplex PCR. A total of 24 stool samples were screened, including 8 containing toxin-producing *C. difficile*, 5 containing non-toxin-producing *C. difficile*, and 11 containing no *C. difficile*. The results are shown in **Figure 1**.

### 2.2 Sample preparation

*C. difficile* strains were inoculated in CCFA medium and incubated in an anaerobic environment at 35°C for 24 hours. *Bacteroides faecis, Clostridium perfringens, Lactobacillus casei*, and *Clostridium* sp*orogenes* strains were inoculated in anaerobic blood agar medium and also incubated in an anaerobic environment at 35°C for 24 hours. *Escherichia coli, Enterococcus faecalis, Klebsiella pneumoniae, Enterobacter inguinalis*, and *Staphylococcus epidermidis* strains were inoculated in Columbia blood agar and incubated in a carbon dioxide environment at 35°C for 24 hours. The pure colonies were isolated from the culture medium and resuspended in 5 mL of tryptic soy broth (TSB) by vortex mixing, followed by grinding against the test tube wall to remove any residual medium.1 mL of the bacterial suspension was centrifuged at 7000 rpm for 2 minutes, the supernatant was discarded, and the pellet was washed three times with sterile water to eliminate impurities and background material. Subsequently, 500 μL of sterile deionized water was added to the pellet, and the suspension was vortex-mixed to resuspend the bacterial cells. The bacterial concentration was adjusted to approximately 10^6-^10^8^ CFU/mL to ensure sufficient Raman signal intensity. A 2 μL aliquot of the suspension was placed on an aluminum-coated Raman microscope slide and air-dried by gently blowing sterile air for 5 minutes to form a homogeneous thin film suitable for spectroscopic analysis.

An appropriate amount of stool sample (approximately 0.5–1 g) was placed into a sterile centrifuge tube, and 10 times the volume of sterile water was added. The mixture was thoroughly homogenized using a vortex shaker until the feces were completely suspended, forming a homogeneous suspension. The suspension was then filtered through a sterile 100 μm filter to remove large particles, such as undigested food and fibers. The filtrate was collected and transferred to a new sterile centrifuge tube. The filtrate was centrifuged at medium speed (3000 × g for 5 minutes) to remove remaining large particles and cellular debris. The supernatant was collected and transferred to a new sterile centrifuge tube for further use. The supernatant was then subjected to high-speed centrifugation (8000 × g for 3 minutes), and the resulting supernatant was discarded, leaving the microbial pellet. This pellet was washed three times with sterile water to remove impurities and background material. Finally, 500 μL of sterile deionized water was added to the microbial sediment, which was vortex-mixed to resuspend the bacterial particles. A 2 μL aliquot of the suspension was then placed on an aluminum-coated Raman microscope slide and air-dried by gently blowing sterile air for 5 minutes, allowing the sample to form a homogeneous thin film suitable for spectroscopic analysis.

### 2.3 Raman data sets, single-cell Raman measurements and preprocessing

A total of 68 independent biological replicates were conducted in this study. To ensure the accuracy of the results, random sampling was employed to collect over 100 single-cell spectra for each sample, yielding a total of 6483 SCRS fingerprints.

Single-cell Raman spectra were acquired using a WITec Alpha300R Raman microspectrometer (WITec, Germany). The instrument was automatically calibrated in silico prior to each measurement, with the calibration peak set to 520 cm^-1^. For the WITec spectrometer, a 532 nm laser was focused onto the sample with a 100× objective(100×/NA = 0.9, ZEISS, Germany) with a power of approximately 15 mW on the sample. Cells were measured with a grating of 600 mm/g, spectral range of 331–3500 cm^−1^, and the spectral center set at 1700 cm^−1^. The Raman acquisition time was 7 s each cell. During measurements of cells that have a larger size compared to the laser spot size, the laser spot was made slightly out-of-focused to cover as much of the whole cell area. The same system was used for all collections, with consistent power settings and integration time to ensure the quality and comparability of the plots.

Preprocessing for the raw Raman spectra included quality control for eliminating abnormally/burnt high-intensity spectra, cosmic ray correction, baseline fitting (polyline fitting, degree at 8, 88 points) and subtraction for autofluorescence removal. The entire spectral area was area normalized so that the sum of all intensities equaled one to account for general instrumentation variability as well as sample and experimental factors without significantly changing the biological content. All model analyses were conducted using Python 3.9. The libraries utilized included, but were not limited to, SciPy for statistical tools, scikit-learn for machine learning models, TensorFlow and PyTorch for deep learning models, and Matplotlib and Seaborn for data visualization.

## 3 Result

### 3.1 *Clostridioides difficile* strain identification

**Figure 2:**
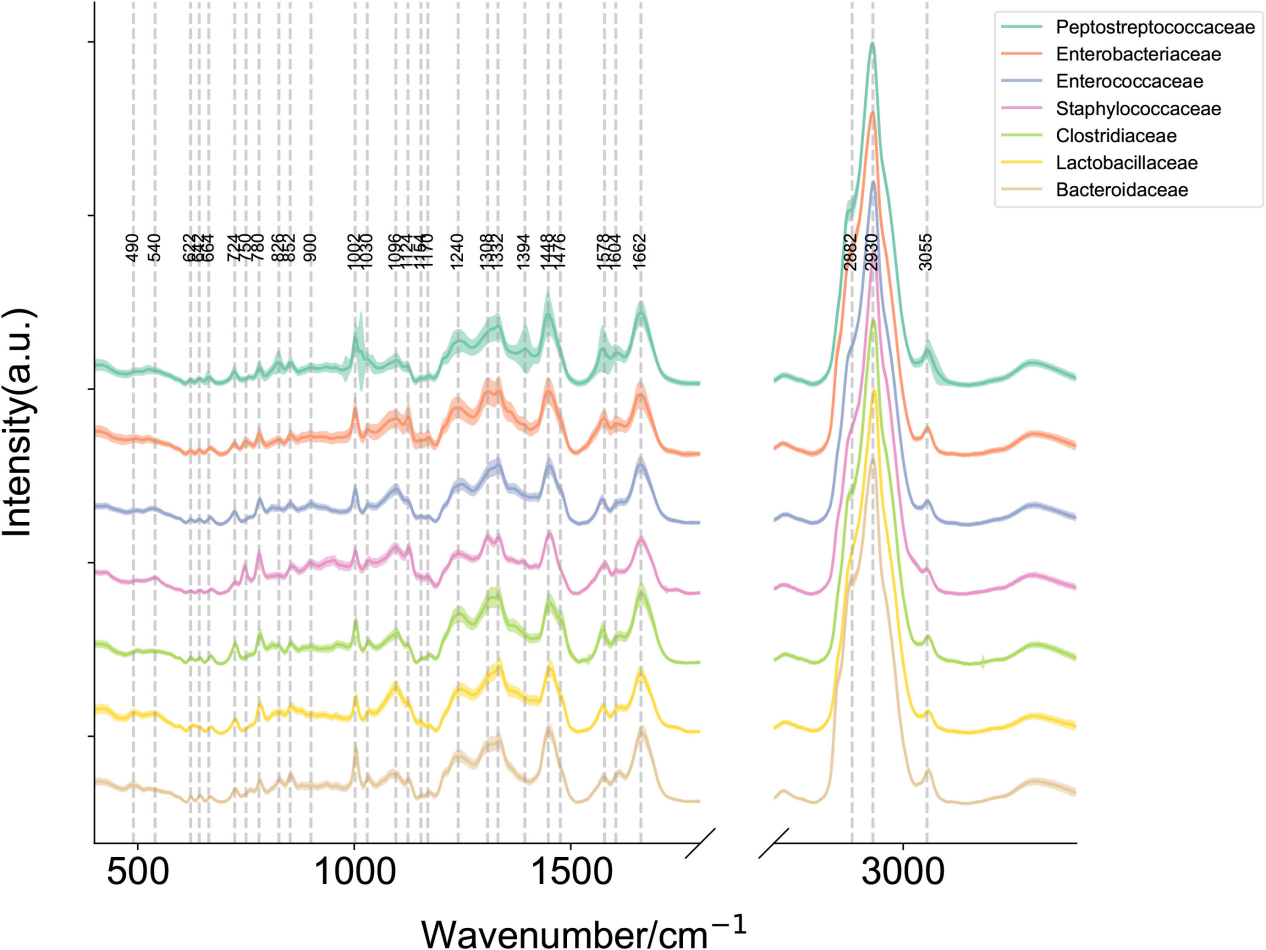
Raman plot for each label with mean and standard deviation. Mean Raman spectra of *Enterobacteriaceae*, *Enterococcaceae*, *Staphylococcaceae*, *Lactobacillaceae*, *Bacteroidaceae*, *Clostridiaceae* and *Peptostreptococcaceae* (Note: *Clostridioides difficile* has been reclassified within the family *Peptostreptococcaceae* under current taxonomic revisions, diverging from its historical classification in *Clostridiaceae*.). The shaded area around each spectrum indicates the standard deviation of the single-cell measurements. (Note: The silent region of Raman spectra (1800–2800 cm^-1^) was omitted from display.).

**Figure 3:**
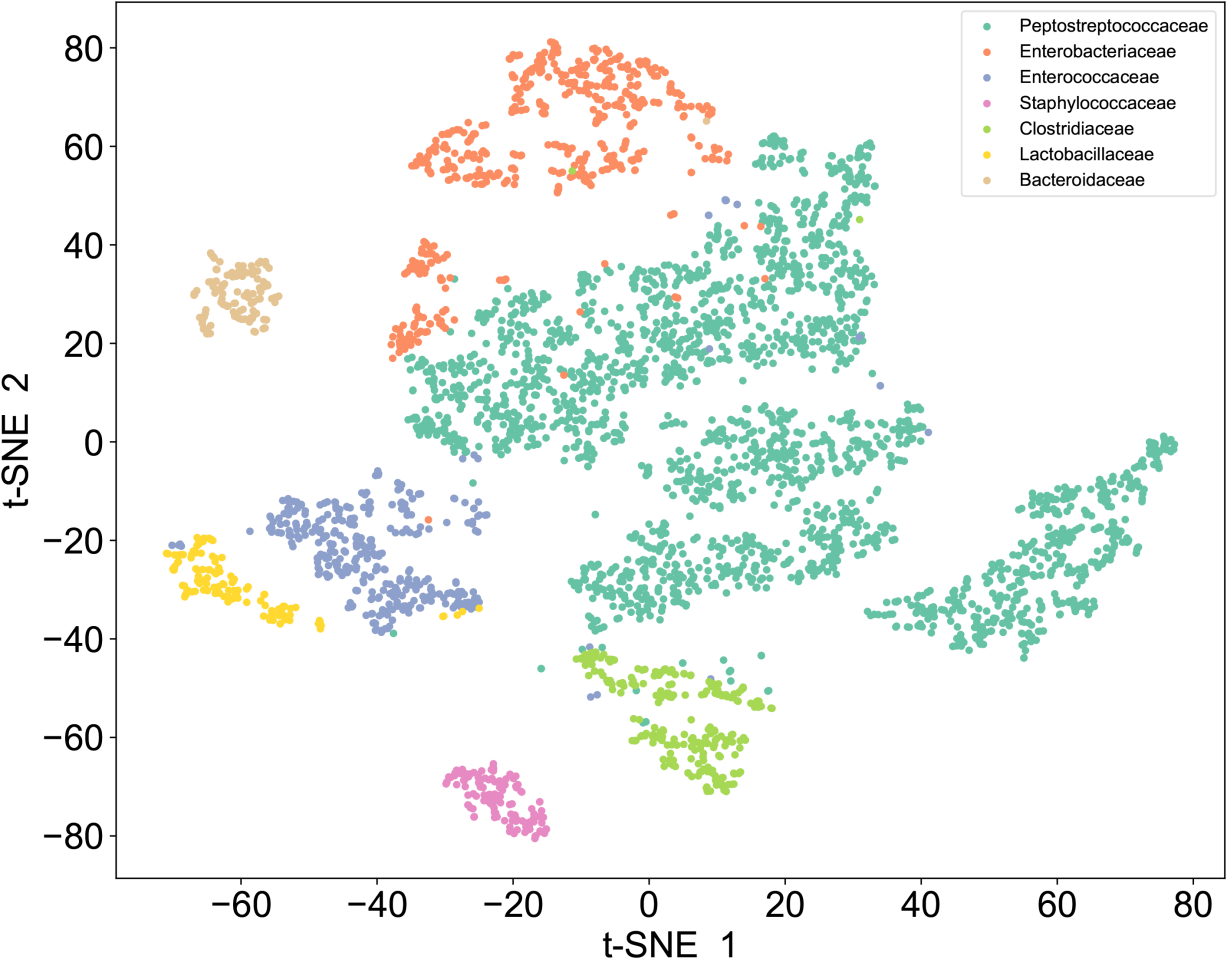
t-SNE of Raman dataset. Unsupervised t-SNE visualization of single-cell Raman spectra reveals seven distinct clusters corresponding to *Enterobacteriaceae, Enterococcaceae, Staphylococcaceae, Lactobacillaceae, Bacteroidaceae, Clostridiaceae*, and *Peptostreptococcaceae*.

**Figure 4:**
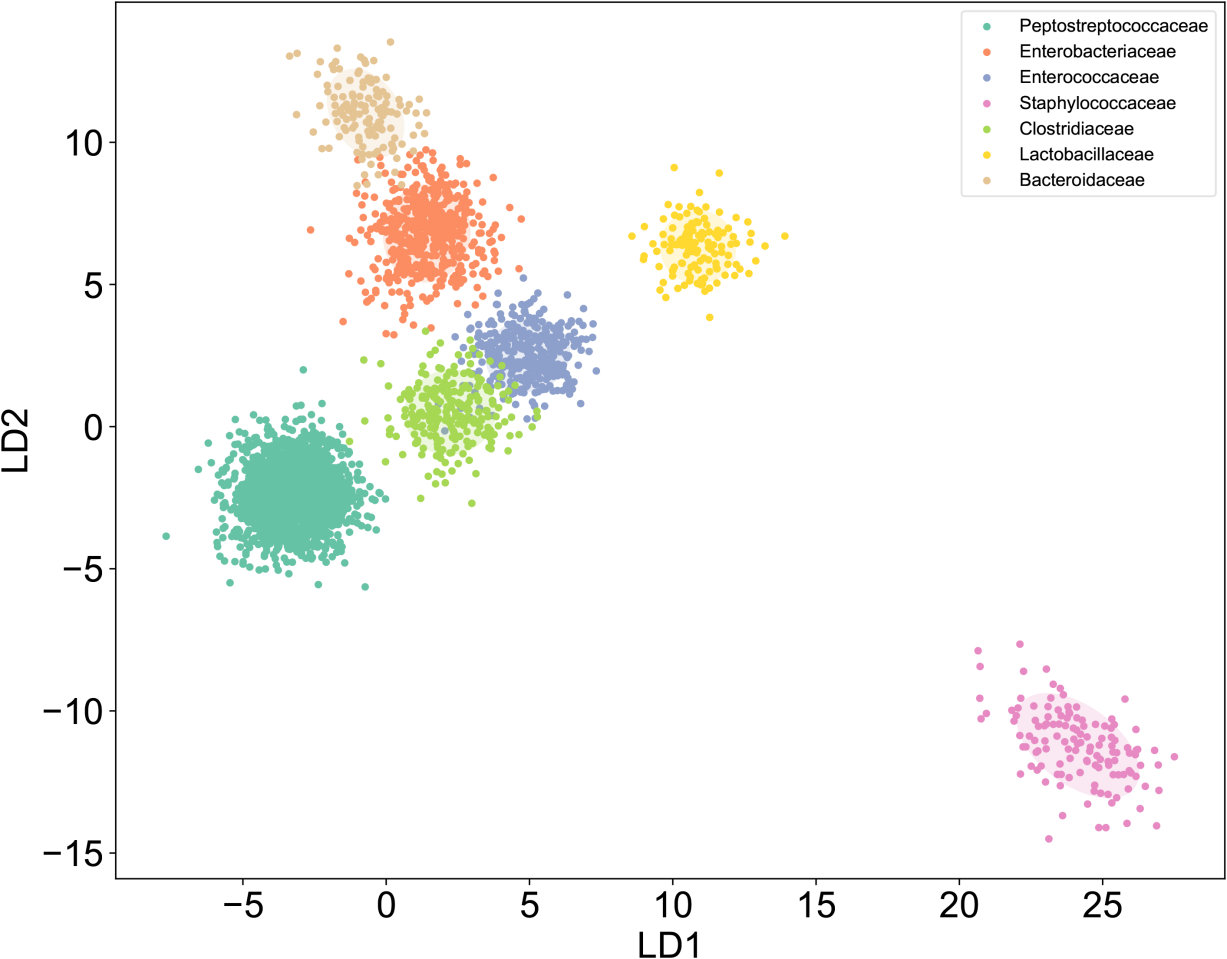
LDA of Raman dataset. Raman spectroscopy coupled with LDA dimensionality reduction showed distinguishable clustering patterns for seven bacterial families: *Enterobacteriaceae, Enterococcaceae, Staphylococcaceae, Lactobacillaceae, Bacteroidaceae, Clostridiaceae*, and *Peptostreptococcaceae.*.

To establish a SCRS-based method for the rapid identification of *C. difficile* directly under the microscope, we first developed a reference SCRS database comprising 12 different bacterial strains representing the most common colonizers of the intestinal tract, including 2 strains of *C. difficile*. To assess the ability of Raman spectroscopy to distinguish *C. difficile* within the species, we included other closely related clostridia, specifically *Clostridium* sp*orogenes* and *Clostridium perfringens*. The list of strains is provided in **Table 1**. More than 100 single cells were measured for each strain, resulting in a total of 1,623 single-cell Raman spectra across the 12 bacterial strains. We grouped the strains into seven categories by family, namely *Enterobacteriaceae, Enterococcaceae, Staphylococcillaceae, Lactobacillaceae, Bacteroidaceae, Clostridiaceae* and *Peptostreptococcacea.*The average Raman spectra for these seven classes of pathogenic bacteria are presented in **Figure 2**. The figure shows that the Raman spectrum of *C. difficile* exhibits multiple characteristic peaks in the range of 1000–1600 cm-¹, especially the peaks near 1002 cm-¹, 1578 cm-¹ and 1662 cm-¹, which may be related to *C. difficile*’s protein secondary structure (e.g., α-helix and β-folding), cell wall components, pigments, and metabolites, and these characteristic peaks distinguish its Raman spectrum from other bacteria (Gelder et al., 2007). The spectral dataset was divided into a training set (80%) and a test set (20%) for classification purposes. Unsupervised t-SNE visualization with low-dimensional observed data showed that the cells of *Enterobacteriaceae, Enterococcaceae, Staphylococcaceae, Lactobacillaceae, Bacteroidaceae, Clostridiaceae* and *Peptostreptococcacea* cells formed obvious clusters in two-dimensional space, which could be effectively differentiated with high intra- and inter-group consistency (**Figure 3**). Linear discriminant analysis (LDA) dimensionality reduction visualization of Raman spectroscopic data demonstrated partial separability among the bacterial families *Enterobacteriaceae, Enterococcaceae, Staphylococcaceae, Lactobacillaceae, Bacteroidaceae, Clostridiaceae*, and *Peptostreptococcaceae* (**Figure 4**).

**Figure 5:**
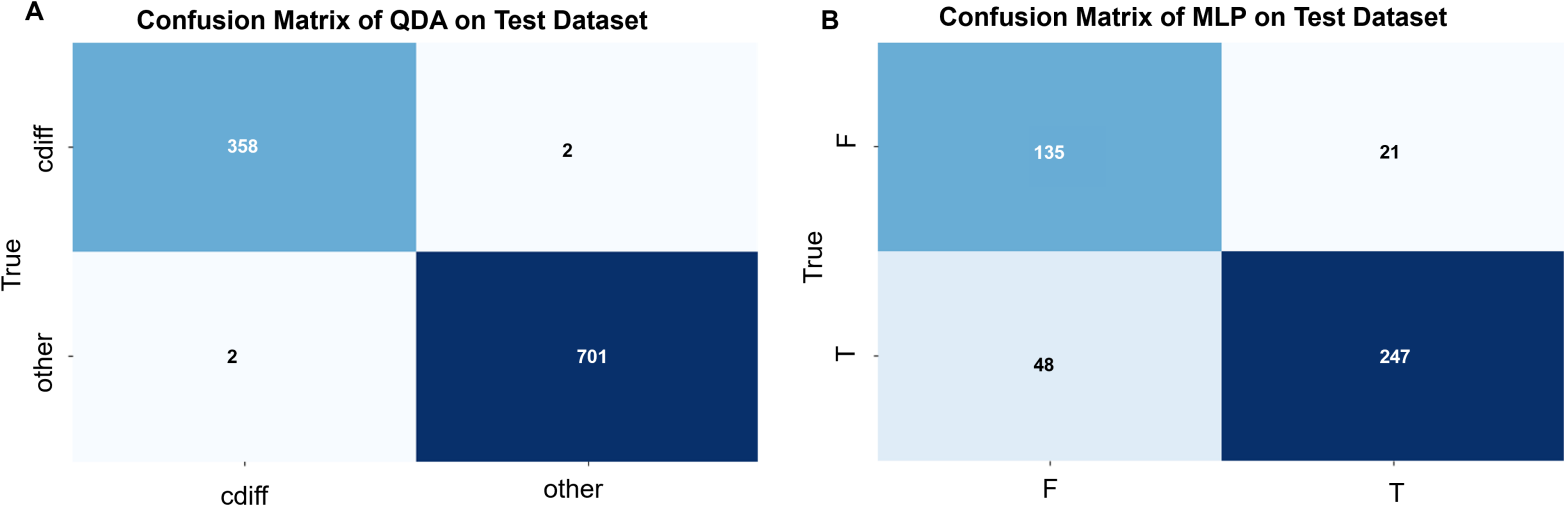
**(A)** Confusion matrix for QDA (Quadratic Discriminant Analysis) modeling (by plot): The *C. difficile* strain identification model was validated using 5 strains of *C. difficile* and 5 strains of other enteric bacteria. A total of 360 single-cell Raman spectra were obtained from the 5 *C. difficile* strains, of which 358 were correctly identified, while 2 spectra were misclassified as other bacteria. Similarly, 703 single-cell Raman spectra were obtained from the 5 strains of other enteric bacteria, of which 701 were correctly identified, and 2 spectra were misclassified as *C. difficile*. The overall prediction accuracy of the model was 0.99. **(B)** Confusion matrix for MLP (Multi-Layer Perceptron) modeling (by plot): The *C. difficile* strain identification model was validated using 1 strain each of toxin-producing and non-toxin-producing *C. difficile*. A total of 295 single-cell Raman spectra were obtained from the toxin-producing *C. difficile*, of which 247 spectra were correctly identified, while 48 spectra were misclassified as non-toxin-producing *C. difficile*. Similarly, 156 single-cell Raman spectra were obtained from the non-toxin-producing *C. difficile*, of which 135 spectra were correctly identified, while 21 spectra were misclassified as toxin-producing *C. difficile*. The overall prediction accuracy of the model was 0.85.

To evaluate the performance of a database identification model constructed based on a single-cell Raman mapping library of pure strains in identifying *C. difficile* strains, we tested it with 10 clinical isolates, including 5 C*. difficile* strains (each derived from a different patient) and 5 other intestinal strains. Additionally, we compared 10 different data analysis methods, all of which demonstrated high accuracy, sensitivity, and specificity (see **Table 2**; **Figure 5A**). This indicates that a database developed from purified strains can be effectively applied to the identification of clinical isolates samples, reliably distinguishing clinically prevalent *C. difficile* strains from other similar strains.

**Table 2:** Comparative evaluation of accuracy, sensitivity, and specificity across computational algorithms for discriminating clinically isolated *Clostridioides difficile* strains from non-*C. difficile* strains.

	Model	Accuracy	Sensitivity	Specificity
0	SVM	1.00	1.00	1.00
1	ANN	1.00	1.00	1.00
2	MLP	1.00	1.00	1.00
3	KNN	1.00	1.00	1.00
4	QDA	1.00	1.00	1.00
5	GRU	1.00	1.00	1.00
6	RF	0.99	0.99	0.99
7	LDA	0.99	0.99	0.99
8	LR	0.99	0.99	0.99
9	NB	0.93	0.93	0.93

Accuracy, sensitivity and specificity of different data algorithms. SVM, Support vector machine; ANN, Multi-Layer Perceptron; MLP, Multi-Layer Perceptron; KNN, K-Nearest Neighbor; QDA, Quadratic Discriminant Analysis; GRU, Gated Recurrent Unit; RF, Random forests; LDA, Linear Discriminant Analysis; LR, Logistic Regression; NB, Naive Bayes.

### 3.2 *Clostridioides difficile* toxin identification

**Figure 6:**
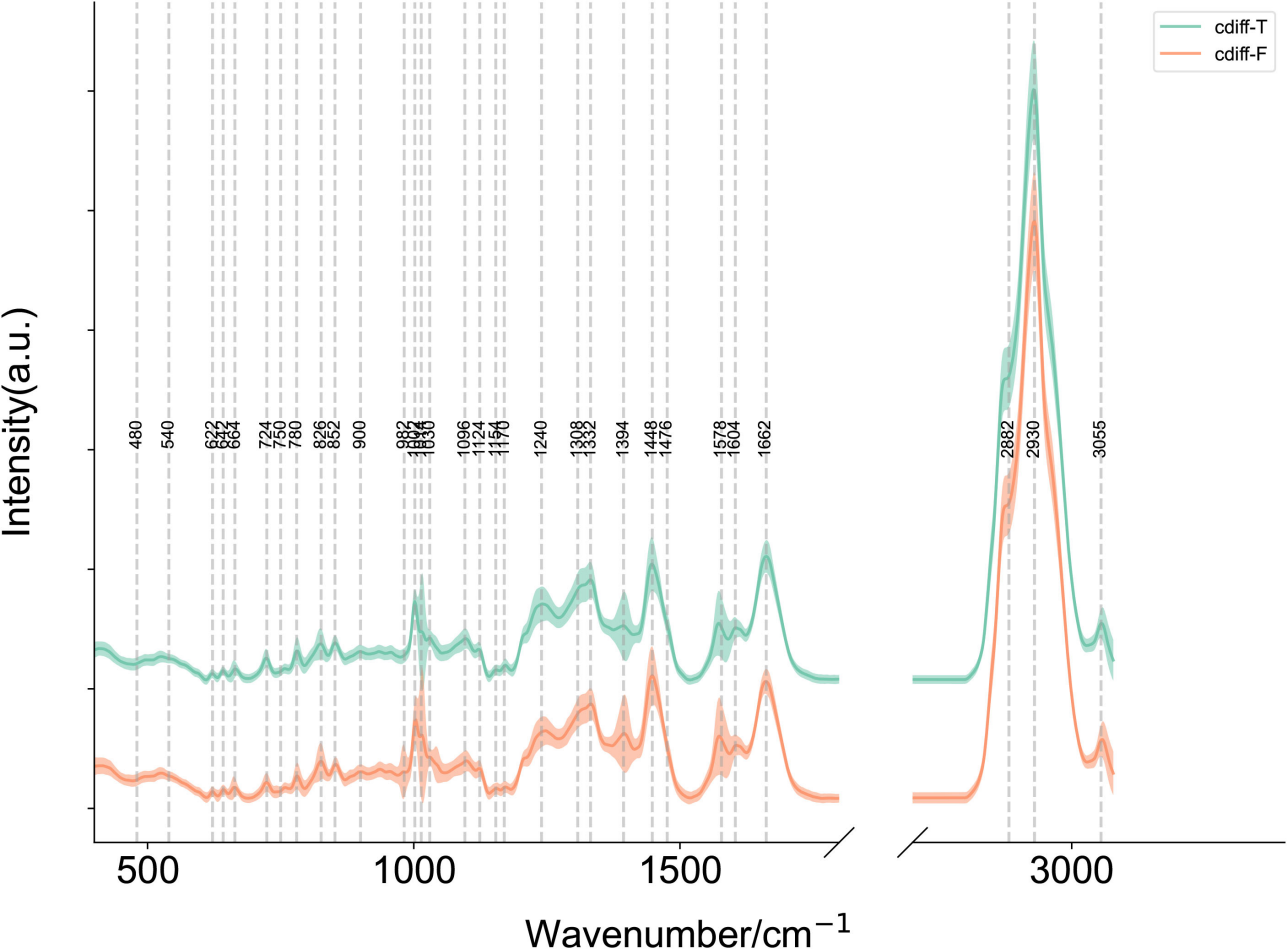
Mean single-cell Raman spectra of toxin-producing (n=5) and non-toxin-producing (n=5) *Clostridioides difficile* strains. Average Raman spectra of cdiff-F (non-toxigenic *C. difficile*), cdiff-T (toxigenic *C. difficile*). The shaded area around each spectrum indicates the standard deviation of the single-cell measurements. (Note: The silent region of Raman spectra (1800–2800 cm^-1^) was omitted from display.).

Since *C. difficile* includes both toxin-producing and non-toxin-producing strains, and its pathogenicity is strictly dependent on the protein toxins produced by the toxin-producing strains, we aimed to develop a SCRS-based method capable of rapidly identifying toxin-producing and non-toxin-producing strains of *C. difficile* directly under the microscope. We first established a reference SCRS database comprising 5 toxin-producing and 5 non-toxin-producing strains of *C. difficile*. The average Raman spectra of these two types of *C. difficile* are presented in **Figure 6**. The figure shows that toxin-producing strains may have different peak intensities in the 500–600 cm-¹ (sulfide-related peaks), 1600–1700 cm-¹ (amide I band), and 2800–3000 cm-¹ (CH_2_/CH_3_ stretching) regions due to the active toxin synthesis pathway and the presence of toxin proteins vibration) regions with different peak intensities.

To evaluate the capability of the SCRS technique in distinguishing between toxin-producing and non-toxin-producing strains of *C. difficile*, we tested 2 clinical isolates of *C. difficile* from different patients, one of which was a toxin-producing strain and the other a non-toxin-producing strain. We compared 9 different data analysis methods, and the results indicated that the Multilayer Perceptron (MLP) exhibited higher specificity and sensitivity compared to the other methods, achieving an identification accuracy of 85% (see **Figure 5B**; **Table 3**).

**Table 3:** Comparative assessment of accuracy, sensitivity, and specificity across computational algorithms for distinguishing toxin-producing from non-toxin-producing *Clostridioides difficile* strains.

	Model	Accuracy	Sensitivity	Specificity
0	MLP	0.85	0.85	0.85
1	ANN	0.83	0.82	0.82
2	RF	0.81	0.8	0.8
3	SVM	0.8	0.82	0.82
4	GRU	0.8	0.79	0.79
5	LDA	0.79	0.8	0.8
6	QDA	0.78	0.73	0.73
7	LR	0.76	0.78	0.78
8	NB	0.75	0.69	0.69

Accuracy, sensitivity and specificity of different data algorithms. MLP, Multi-Layer Perceptron; ANN, Multi-Layer Perceptron; RF, Random forests; SVM, Support vector machine; GRU, Gated Recurrent Unit; LDA, Linear Discriminant Analysis; QDA, Quadratic Discriminant Analysis; LR, Logistic Regression; NB, Naive Bayes.

### 3.3 *In situ* identification of clinical stool samples

**Figure 7:**
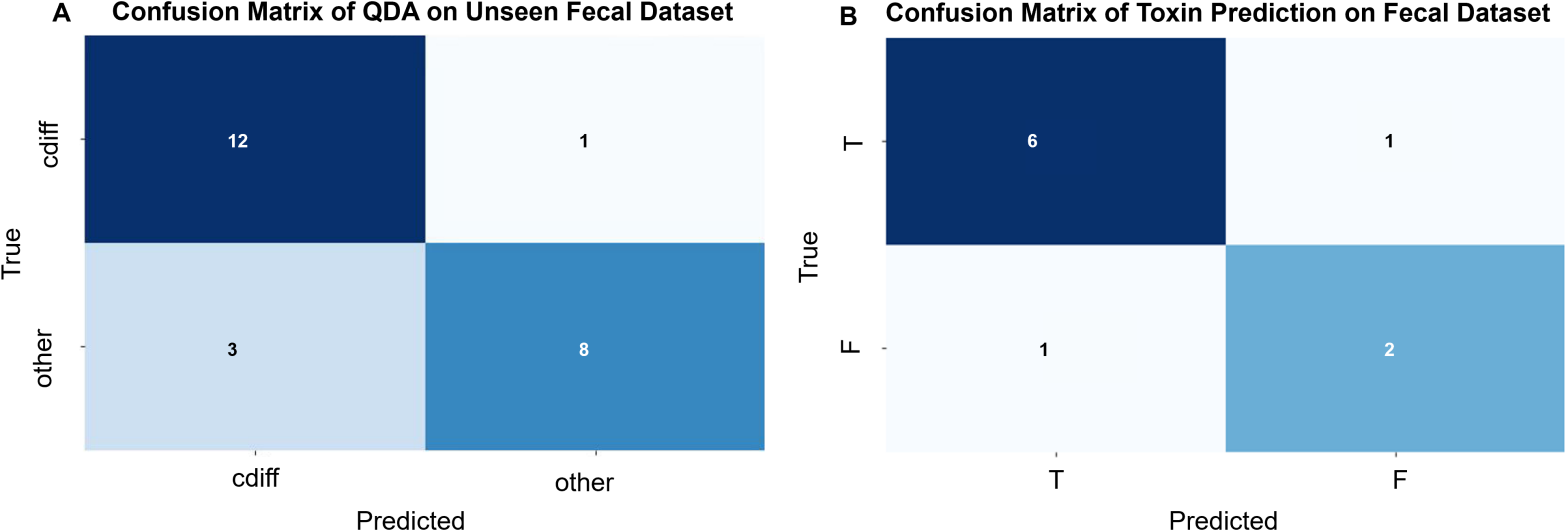
**(A)** Confusion matrix for the QDA (Quadratic Discriminant Analysis) model (by sample): Validation of the *C. difficile* strain identification model was performed using 24 original stool samples. Among the 13 *C. difficile*-positive stool samples, 12 were correctly identified, while 1 was misclassified as *C. difficile*-negative. Of the 11 *C. difficile*-negative stool samples, 8 were correctly identified, while 3 were misclassified as *C. difficile*-positive. The overall predictive accuracy of the model was 0.83. **(B)** Confusion Matrix of Toxin Prediction on Fecal Dataset (by sample):10 original fecal samples containing *C. difficile* were used to validate the *C. difficile* strain identification model. Of the 7 fecal samples containing toxin-producing *C. difficile*, 6 were correctly identified, while 1 was misclassified as containing non-toxin-producing *C. difficile*. Among the 3 fecal samples containing non-toxin-producing *C. difficile*, 2 were correctly identified, while 1 was misclassified as containing toxin-producing *C. difficile*. The overall predictive accuracy of the model was 0.80.

To evaluate the database model’s ability to identify *C. difficile* strains in raw stool samples and distinguish between toxin-producing and non-toxin-producing strains, we tested 24 raw clinical stool samples. These samples were validated by culture and PCR methods, with 13 containing *C. difficile* and 11 not containing *C. difficile*, each from a different patient. Due to the presence of numerous stool strains, some closely related to *C. difficile* and easily misidentified, we applied a stringent confidence threshold, requiring the model to have a probability greater than 0.99 to classify a profile as *C. difficile*, all intermediate probabilities were classified as other. By comparing various data analysis methods, we found that the best model was Quadratic Discriminant Analysis (QDA), which outperformed the deep learning model, with a prediction accuracy of 0.83 (see **Figure 7A**). There was 1 instance where *C. difficile* was misidentified as other, and 3 instances where other strains were misidentified as *C. difficile*.

Among the correctly identified stool samples, *C. difficile* was present in 12 samples; however, 2 samples were excluded from this prediction due to a low number of validated maps. Consequently, a total of 10 stool samples were used for *C. difficile* toxin modeling, comprising 7 cases with toxin-producing *C. difficile* and 3 cases with non-toxin-producing *C. difficile*. The results indicated that, out of the 10 stool samples, 8 were accurately predicted and 2 were incorrectly predicted, yielding an accuracy of 80.0% (see **Figure 7B**).

## 4 Discussion

*C. difficile* represents a significant pathogen in nosocomial infections, with an escalating incidence of diarrhea and associated illnesses attributed to this bacterium observed globally in recent years (Carvalho et al., 2022). This rise has contributed to substantial morbidity and mortality worldwide. The pathogenesis of CDI is intrinsically linked to the secretion of proteotoxins by toxigenic strains of *C. difficile* (Slimings and Riley, 2014). Consequently, prompt and accurate detection of these toxigenic strains is crucial, not only for reducing the overdiagnosis of CDI but also for enhancing the accuracy of its diagnosis and the effectiveness of preventative strategies.

Current laboratory methodologies for CDI diagnosis encompass a variety of techniques, including stool *C. difficile* culture, cell culture cytotoxin assay, immunological toxin assays, glutamate dehydrogenase (GDH) assay, and nucleic acid amplification tests, among others. *C. difficile* is a strictly anaerobic bacterium that requires stringent growth conditions, specialized equipment and media, slow growth, prolonged incubation periods, and complex, cumbersome procedures, all of which contribute to its low sensitivity. The cytotoxicity test, regarded as the “gold standard” for laboratory diagnosis of CDI (Xu et al., 2019), presents challenges due to its complicated, time-consuming, and costly nature, rendering it impractical for routine use in clinical laboratories. The enzyme immunoassay (EIA) method is simple to perform, rapid, and demonstrates high specificity (>90%). However, its sensitivity is limited (ranging from 39% to 76%) and is influenced by specimen characteristics and prior empirical clinical treatments (Perelle et al., 1997; Carter et al., 2007). Additionally, it is associated with a high rate of false positives and instability of antibodies (Sakamoto et al., 2018). The GDH assay, noted for its rapidity and cost-effectiveness, also does not provide information regarding toxin production and is typically employed as an initial screening tool (Crobach et al., 2016). The polymerase chain reaction (PCR) method offers excellent specificity and high sensitivity, alongside rapid detection times. However, the complexity of its operational procedures, the need for expensive equipment, and the requirement for skilled personnel limit its application, particularly in settings with limited resources. Raman spectroscopy can provide a “chemical fingerprint” of *C. difficile* within 1 hour, enabling rapid identification of *C. difficile* for initial screening and classification. At the same time, in combination with PCR technology, the accuracy of Raman results can be verified, which is suitable for clinical diagnosis (Choi and Schlücker, 2024).

SCRS is a “total biometric fingerprinting” technique that enables rapid, non-destructive characterization and identification of individual cells without the need for additional markers. It also facilitates predictions at both the genotypic and phenotypic levels. Raman spectroscopy is non-destructive, in contrast to MALDI-TOF MS, which requires additional sample preparation steps, such as the addition of formic acid and substrates, potentially leading to alterations in analyte signal intensity (AlMasoud et al., 2021). Furthermore, MALDI-TOF MS typically relies on pure bacterial cultures grown on solid media and cannot differentiate between toxin-producing and non-toxin-producing *C. difficile* strains. Compared to techniques such as sequencing and molecular labeling, Raman spectroscopy can be more easily applied to the detection of new strains because it does not require the use of specifically designed labels. In addition, the single-cell nature of Raman spectroscopy enables rapid identification in the early stages of infection. Its highly automated nature simplifies the diagnostic process and interpretation of results, making it particularly suitable for use in environments with limited training resources or restricted clinical conditions.

For instance, Wang et al. employed Raman spectroscopy in conjunction with neural networks to identify *archaeobacteria* and utilized a convolutional neural network (CNN) for the classification of *Enterobacteriaceae* species, achieving an accuracy of 97.2% (Wang et al., 2020). Rebrošová et al. applied Raman spectroscopy for the swift identification of *Staphylococcus* species (Rebrošová et al., 2017), while Jiabao Xu and colleagues utilized the technique to identify 94 clinical isolates with a perfect accuracy rate of 100%, also providing precise diagnoses for 7 original urine samples (Carvalho et al., 2022). Furthermore, Ziyu Liu et al. utilized SCRS to classify 6 strains of respiratory pathogens, attaining accuracies ranging from 93% to 100%, and achieved more than 80% accuracy with clinical samples (Liu et al., 2023). Kloß et al. demonstrated the direct application of Raman spectroscopy for pathogen identification in ascites, where the results exhibited 97.7% and 83.6% correctness at the genus and species levels, respectively (Kloß et al., 2015). In the current study, Raman spectroscopy combined with a neural network was used to identify clinical isolates of *C. difficile* with 100% accuracy, and provided an 83% accuracy rate in raw stool samples, thus highlighting the significant potential of Raman spectroscopy in clinical diagnostics.

Additionally, Raman spectroscopy has been employed to characterize microbial virulence factors, including mechanisms of antimicrobial resistance. For instance, Zhou et al. utilized surface-enhanced Raman scattering (SERS) to successfully differentiate between wild-type and drug-resistant strains of *Escherichia coli* (Zhou et al., 2015). Jing-Wen Lyu and colleagues integrated SERS spectroscopy with a deep learning algorithm to accurately differentiate between 121 clinically isolated strains of *Klebsiella pneumoniae* (PRKP, CRKP, and CSKP) with varying resistance profiles (Lyu et al., 2023). Jiayue Lu et al. developed a CNN for the rapid identification of antimicrobial resistance genes (ARGs), high virulence coding factors, and resistance phenotypes in 71 strains of *Klebsiella pneumoniae*, using raw Raman spectral data (Lu et al., 2022). Similarly, Shu Wang et al. applied a label-free SERS method to distinguish between 60 strains of *Staphylococcus aureus*, including methicillin-sensitive (MSSA) and methicillin-resistant (MRSA) strains, achieving an identification accuracy of 100% (Wang et al., 2021). The findings from these studies indicate that SCRS, when coupled with deep learning algorithms, enhances the capability to distinguish between toxin-producing and non-toxin-producing strains of *C. difficile*, with classification accuracies of 85% in isolated samples and 80% in clinical samples. These results suggest the potential of Raman spectroscopy as a rapid detection method for identifying toxin-producing strains of *C. difficile*, underscoring its utility in clinical diagnostics. The results of Punjabi et al. also indicated that Raman spectroscopy facilitates rapid detection (POCT) in a simple and cost-effective manner, thereby making it a valuable tool for immediate care (Sin et al., 2014; Guo et al., 2019; Punjabi et al., 2022).

The utility of Raman spectroscopy in the identification of *C. difficile* and its toxins has been previously limited (Koya et al., 2018; Koya et al., 2019; Hassanain et al., 2021). However, the current study employed Raman spectroscopy for the *in situ* identification of *C. difficile* within raw stool samples and for distinguishing between toxin-producing and non-toxin-producing strains. This application provides valuable data that could inform the use of this technique in diagnosing *C. difficile* infections. In this investigation, the accuracy of Raman spectroscopy in identifying toxin-producing versus non-toxin-producing strains of *C. difficile* ranged from moderate to high. Future research will involve a larger sample size and the implementation of increasingly sophisticated methods for variable analysis to enhance the diagnostic accuracy of this approach.

## Data Availability

The raw data supporting the conclusions of this article will be made available by the authors, without undue reservation.
